# Optimizing the First Step of the Biocatalytic Process for Green Leaf Volatiles Production: Lipase-Catalyzed Hydrolysis of Three Vegetable Oils

**DOI:** 10.3390/ijms241512274

**Published:** 2023-07-31

**Authors:** Eva Faillace, Virginie Brunini-Bronzini de Caraffa, Magali Mariani, Liliane Berti, Jacques Maury, Sophie Vincenti

**Affiliations:** Laboratoire de Biochimie et Biologie Moléculaire Végétales, Campus Grimaldi, Université de Corse, CNRS UMR6134 SPE, BP52, 20250 Corte, France; faillace_e@univ-corse.fr (E.F.); brunini_v@univ-corse.fr (V.B.-B.d.C.); mariani_m@univ-corse.fr (M.M.); berti_l@univ-corse.fr (L.B.)

**Keywords:** lipase, hydrolysis, sunflower oil, hempseed oil, linseed oil, response surface methodology

## Abstract

Green leaf volatiles (GLVs), including short chain volatile aldehydes, are widely used in the flavor and food industries because of their fresh aroma. To meet the growing demand for natural GLVs with high added value, the use of biocatalytic processes appears as a relevant application. In such processes, vegetable oils are bioconverted into GLVs. First, the triacylglycerols of the oils are hydrolyzed by a lipase. Then, the free polyunsaturated fatty acids are converted by a lipoxygenase. Finally, volatile C6 or C9 aldehydes and 9- or 12-oxoacids are produced with a hydroperoxide lyase. Optimization of each biocatalytic step must be achieved to consider a scale-up. In this study, three oils (sunflower, hempseed, and linseed oils) and three lipases (*Candida rugosa*, *Pseudomonas fluorescens*, and *Rhizomucor miehei* lipases) have been tested to optimize the first step of the process. The experimental design and response surface methodology (RSM) were used to determine the optimal hydrolysis conditions for each oil. Five factors were considered, i.e., pH, temperature, reaction duration, enzyme load, and oil/aqueous ratio of the reaction mixture. *Candida rugosa* lipase was selected as the most efficient enzyme to achieve conversion of 96 ± 1.7%, 97.2 ± 3.8%, and 91.8 ± 3.2%, respectively, for sunflower, hempseed, and linseed oils under the defined optimized reaction conditions.

## 1. Introduction

Green leaf volatile (GLV) compounds, including short-chain aldehydes, alcohols, and esters, are important contributors to the characteristic flavors of fruits, vegetables, and green leaves. They are naturally produced in higher plants by the lipoxygenase pathway [1], which is activated during different stages of plant development [2,3] and in response to biotic or abiotic stresses [4,5,6].

The lipoxygenase pathway involves the oxidation of lipids to produce signaling and defense molecules called phytooxylipins, including GLVs [4,7]. First, membrane phospholipids and galactolipids are hydrolyzed by phospholipases and galactolipases [8]. Afterwards, lipoxygenase (LOX) catalyzes the dioxygenation of free polyunsaturated fatty acids (PUFAs), like linoleic and linolenic acids, to form corresponding hydroperoxides (HPO) [1,9]. In the last step, HPOs are converted into C6- or C9-aldehydes and 12- or 9-oxoacids by the action of hydroperoxide lyase (HPL) [10,11]. Compounds produced by the action of HPL may be of industrial interest. The oxoacids can be used in industries for the synthesis of biopolymer precursors [12,13]. The C6- or C9-aldehydes, like hexanal or (3*Z*)-nonenal, are part of GLVs and are responsible for the fresh smell of green leaves and cut grass, referred to as “green note” [14]. Thus, they are widely used in the food and cosmetic industries for their flavoring properties. They can be added to food components to restore the natural fresh aroma of fruits or vegetables lost during industrial processing or sterilization processes. Due to their antibacterial and antifungal activities [15], these molecules are also used to extend the shelf life of food products.

The industrial production of these molecules of interest is therefore an important challenge. Currently, the global market for flavors and fragrances is worth USD 18.7 billion [16]. These molecules are mainly produced by chemical synthesis, which is environmentally unfriendly. Nowadays, consumers show a strong preference for natural flavors. Unfortunately, the extraction of natural GLVs from raw plants leads to low yields. So, given the high demand for natural flavors, the development of biocatalytic processes using enzymes of the lipoxygenase pathway seems relevant [17]. In such processes, vegetable oils are bioconverted into GLVs and oxoacids through the actions of lipases, LOXs, and HPLs. In order to realize a process for the efficient production of industrial interest compounds, the optimization of each step is necessary.

**Figure 1:**
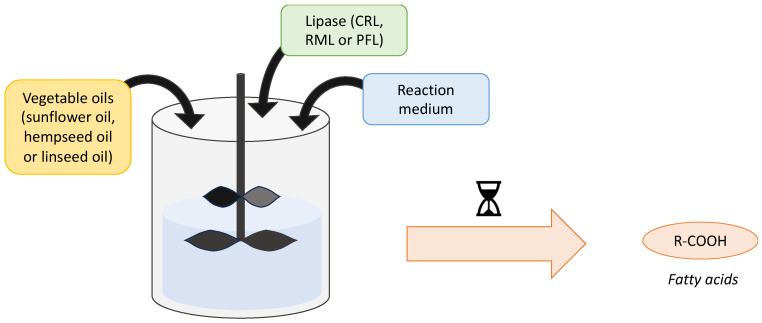
Scheme of the biocatalytic hydrolysis process of vegetable oils to release fatty acids.

In the present study, the optimization of the first reaction step of the process, i.e., the lipolysis of a vegetable oil, chosen according to its lipid profile, is investigated (Figure 1). The choice of the oil according to its fat composition will allow for selecting the class of HPO synthetized and, thus, the class of GLVs produced. A hydrolysis reaction to convert a lipid complex into PUFAs can be performed using a chemical process (with an acid or base) under drastic conditions that require the use of a large amount of solvents. Instead of being produced by chemical synthesis, these compounds can be obtained by enzymatic hydrolysis using a lipase under mild reaction conditions [18,19]. Indeed, biocatalytic processes can be less energy-intensive than synthetic chemical processes and consume fewer raw materials [20]. Lipases (triacylglycerol acylhydrolases, EC 3.1.1.3) are known to catalyze fats and oils and to release a large amount of free fatty acids, monoacylglycerols, diacylglycerols, and glycerol from oil. In addition to their catalytic polyvalence and their good stability, lipases are able to accept a wide diversity of substrates and to tolerate many forms (liquid, solid as native enzymatic powder, and immobilized enzymes) [21]. The use of lipases in the biocatalytic process can be relevant because these biocatalysts have already been successfully use in biotechnological applications for the synthesis of biopolymers and biodiesel and the production of enantiopure, agrochemicals, flavor compounds, and pharmaceuticals [22]. The chemo-, regio-, and enantiospecific behaviors of these enzymes are therefore significant advantages in industrial processes.

We propose to test different lipases and vegetable oils and to perform a screening of the hydrolysis conditions for each. To evaluate the effect of different factors (pH, temperature, duration reaction, enzyme load, and oil/aqueous ratio of the mixture) on the hydrolysis reaction, a response surface methodology (RSM) is employed. This methodology helps identify the best factor levels to optimize the reaction conditions and to maximize the hydrolysis rate. Thus, for each oil, the most effective lipase and the best hydrolysis conditions are determined.

## 2. Results and Discussion

### 2.1. Lipid Profile of Oils

The three oils selected (sunflower, hempseed, and linseed) were analyzed by gas chromatography. The FA composition of each oil is shown in Table 1.

Sunflower oil contains linoleic acid (55.3%), oleic acid (33.3%), and saturated FA (9.5%). Hempseed oil has the particularity of having a high content of PUFAs, such as 56.6% linoleic acid and 19.0% linolenic acid, and low contents of oleic acid (4.4%) and saturated FA (12.7%) [13,23]. Selected linseed oil is rich in linolenic acid (53.0%), oleic acid (20.6%), linoleic acid (15.5%), and saturated FA (9.5%). In a biocatalytic process, hexanal or (3*Z*)-nonenal (responsible for the smell of cut grass, apples, or cucumber-like scents) are obtained from linoleic acid, while (3*Z*)-hexenal or (3*Z*,6*Z*)-nonadienal (responsible for the odor of green leaves, grass, and melon) are obtained from linolenic acid.

The saponification value of the sunflower, hempseed, and linseed oils measured is, respectively, 195.8 mgKOH.g^−1^, 193.4 mgKOH.g^−1^, and 186.6 mgKOH.g^−1^. The total amount of FAs for each of the oils is therefore 3.49 mmol FAs.g^−1^ for sunflower oil, 3.45 mmol FAs.g^−1^ for hempseed oil, and 3.33 mmol FAs.g^−1^ for linseed oil.

### 2.2. Determination of Optimum pH and Optimum Temperature of Lipases

**Figure 2:**
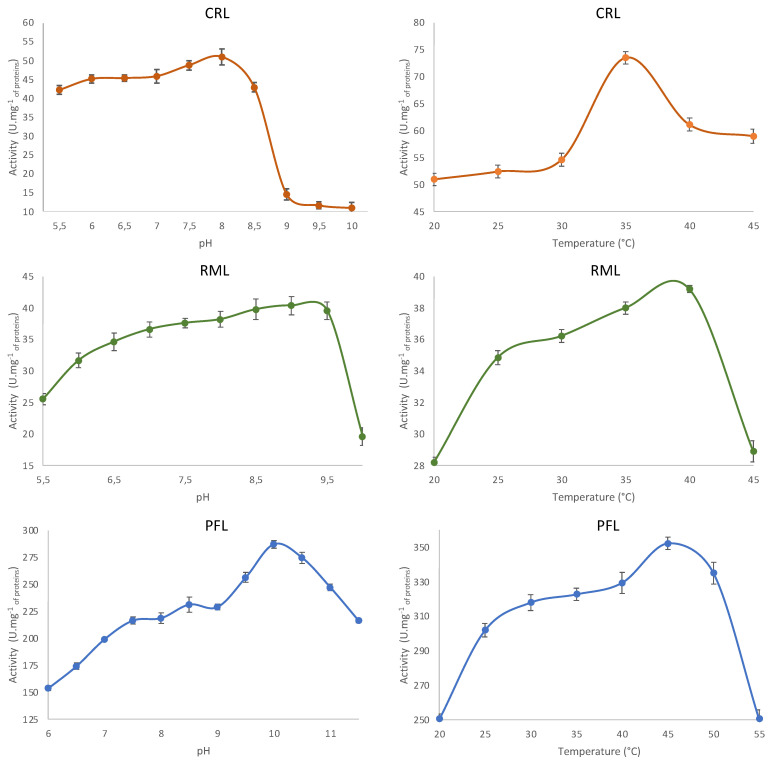
Influence of pH and temperature on the specific lipase activities of CRL, RML, and PFL.

The influence of the pH and temperature on the lipase activity of the three selected lipases (CRL, RML, and PFL) is presented in Figure 2 below.

The optimum pH values for CRL, RML, and PFL are 8, 9, and 10, respectively. The pH values described in the literature are generally between 6 and 11 [24,25,26]. Our results show that for CRL and RML, the activity is preserved at more than 80% over a pH range of 5.5 to 8.5 for CRL and 6.5 to 9.5 for RML. The optimal temperature of the enzymes is 35 °C for CRL, 40 °C for RML, and 45 °C for PFL. CRL retains over 70% of its activity over a temperature range from 25 to 45 °C. RML retains 85% of its activity over a range from 25 to 40 °C, while PFL retains over 70% of its activity over a pH range from 7 to 11 and over a temperature range from 20 to 55 °C. These lipases are stable over a wide pH and temperature range.

### 2.3. Optimization of the Hydrolysis Rate Using a Central Composite Design

The optimization of the hydrolysis reaction of the selected vegetable oils by lipases was performed using an RSM. A central composite design was used to evaluate the effect of the pH (H) and the temperature (T) on the hydrolysis rate over several hours. The results obtained from 39 experimental runs carried out according to the central composite design are summarized in the Supplementary Materials, Table S1. The hydrolysis rate varies from 1.4% to 93.7% for sunflower oil, from 0% to 100% for hempseed oil, and from 1.9% to 100% for linseed oil.

A regression analysis was performed on the experimental data. Then, a second-order polynomial equation was fitted to the measured values of the hydrolysis rate. Thus, the equations shown in Table 2. model the relationship between the factors (pH and temperature) and the hydrolysis rate.

R^2^ coefficients of the models are, respectively, for CRL, RML, and PFL 96.21%, 88.43%, and 89.83% for sunflower oil; 93.88%, 89.94%, and 91.99% for hempseed oil; and 92.90%, 91.20%, and 92.42% for linseed oil. The adjusted R^2^ shown in Table 2 indicates that more than 86.68% of the variability in the hydrolysis rate could be explained by the model. These values of R^2^ reveal a satisfactory adjustment of the models to the experimental data, so these models can be used for the analysis and prediction of the hydrolysis rate.

An analysis of variance (ANOVA) was carried out to assess the significance of the fit of the response surface model for the hydrolysis rate. A *p*-value <0.05 was considered to be significant. The models are shown in Table 3.

The RSM analysis for the sunflower oil lipolysis with CRL, RML, and PFL showed a *p*-value lower than 0.05 for each of the linear terms H and T and the quadratic terms H^2^ and T^2^, meaning both factors have a significant influence on the hydrolysis rate. The interaction term H × T is significant only with CRL action ((a) in Table 3).

Concerning the hempseed oil lipolysis with CRL, RML, and PFL, the linear terms H and T and the quadratic terms H^2^ and T^2^ exhibit a significant influence on the hydrolysis rate. The interaction term H × T is only significant for the RML and PFL action ((b) in Table 3).

Regarding the RSM analysis of linseed oil, the linear term H and the quadratic term H^2^ are significant for the action of the three lipases, while the linear term T and the quadratic term T^2^ have a significant influence on the hydrolysis rate with CRL and PFL action. The interaction term H × T reveals a statistically significant influence only with the RML and PFL action ((c) in Table 3).

**Figure 3:**
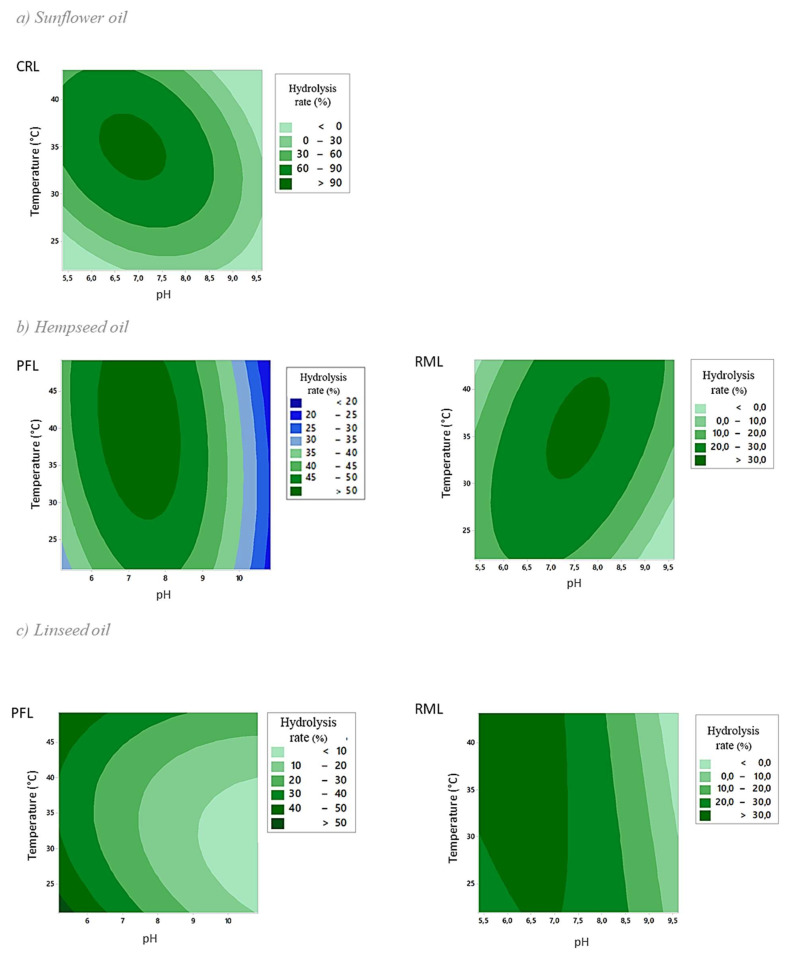
Contour plots of the hydrolysis rate (%) after lipolysis on sunflower oil (**a**), hempseed oil (**b**), and linseed oil (**c**), as a function of the temperature (T) and pH (H) for CRL, PFL, and RML.

The effects of cross-term product factors on the rate of hydrolysis can be examined using contour plots. Only significant interactions are retained in Figure 3. Other contour plots, in addition to three-dimensional (3D) response surface plots, are shown in the Supplementary Data, Figures S1 and S2, respectively.

The lipase that achieves the best lipolysis seems to be CRL, with a hydrolysis rate over 90% (Figure 3a) for sunflower oil, while hydrolysis rates over 50% and 30% are barely reached, respectively, with the action of PFL (Figure 3c) and RML (Figure 3b) for linseed and hempseed oils.

For sunflower and hempseed oils, high hydrolysis rates are measured when the pH is around 7 and for temperatures around 30 °C. Concerning the temperature, when the temperature is low (<25 °C), lower hydrolysis rates are measured, whereas the highest hydrolysis rates are measured between 30 and 40 °C (Figure 3a,b). This is explained by the fact that the probability of collision between the substrate and the enzyme increases with the temperature [27,28]. The range of temperatures for which high rates are obtained is wider for linseed oil than for sunflower and hempseed oils, especially concerning the action of RML, where only the pH showed a significant influence on the hydrolysis reaction (Table 3). Concerning the pH, for linseed oil, the maximum hydrolysis rates are measured when the pH is more acidic, regardless of the temperature tested (Figure 3c). Globally, the data show that regardless of the oil or the lipase, an alkaline pH leads to low hydrolysis rates (Figure 3). The parameter with the greatest impact on the reaction seems to be pH, which needs to be close to neutral or acidic in order to obtain a high hydrolysis rate. Interface quality is essential to obtain a high hydrolysis rate. Given the essential role of the interface, the optimum pH and the optimum temperature of the enzymes could differ over several hours and also from one oil to another [29,30,31].

The RSM approach provides optimal pH and temperature values to maximize the hydrolysis rate and predicts the maximum value of the hydrolysis rate with these optimal conditions. Then, the predicted hydrolysis rate is subject to experimental validation. The results are shown in Table 4.

For sunflower oil, the maximum hydrolysis rate is reached at 35 °C and at a pH of 7 with the action of CRL (96.4 ± 1.8%) and RML (38.4 ± 2.3%) and at 34 °C and at a pH of 5.2 with PFL action (20.2 ± 1.5%). The hydrolysis rates have a standard deviation of 0.2%, 3.7%, and 2.5%, respectively, with the predicted hydrolysis rates (Table 4, sunflower oil).

For hempseed oil, the highest hydrolysis rates are reached at 35 °C and at a pH of 7.5 with CRL action (87.9 ± 3.6%), at 36 °C and at a pH of 7.6 with RML action (40.3 ± 3.2%), and at 35 °C and at a pH of 7.5 with PFL action (48.7 ± 1.9%). The standard deviation is 1.4%, 27.9%, and 8.4% with the predicted values, respectively (Table 4, hempseed oil).

The lipolysis of linseed oil is maximal at 30 °C and at a pH of 6 with CRL action (93.0 ± 3.8%), at 43 °C and at a pH of 6 with the action of RML (39.0 ± 1.5%), and at 21 °C and at a pH of 5.2 with PFL action (56.1 ± 2.7%). The standard deviation is 2.7%, 7.4%, and 1.3% with the predicted values, respectively (Table 4, linseed oil).

Under optimal conditions of pH and temperature, the best yield is obtained with CRL for the three selected oils. CRL is described as a lipase acting on all three positions of triacylglycerol, unlike the other two lipases, which would explain its ability to release more FAs over the same period. For RML and PFL, a hydrolysis rate of over 50% has rarely been described, regardless of the medium used (heterogeneous solvent-free medium or heterogeneous medium with organic solvent) [28,32].

Finally, our results show that CRL is the most efficient lipase on the three oils considering their hydrolysis rates. Thus, this lipase was selected for the next experiments.

### 2.4. Optimization of the Hydrolysis Reaction Using a Box–Behnken Design

The optimization of the hydrolysis reaction of the three different vegetable oils with the CRL is carried out using an RSM at the optimum pH and temperature previously determined. A Box–Behnken design is used to investigate the influence of the duration time (D), the enzyme load (E), and the oil/aqueous ratio of the mixture (O) on the hydrolysis rate. The results obtained from 45 experimental runs performed according to the Box-Behnken design are summarized in the Supplementary Materials, Table S2. Hydrolysis rates ranged from 32.4% to 96% for sunflower oil, 22.6% to 100% for hempseed oil, and 47.8% to 100% for linseed oil.

A regression analysis was performed on the experimental data. The second-order polynomial equation was fitted to the measured values of the hydrolysis rate. Thus, the equations shown in Table 5 model the relationship between the factors (duration time, enzyme load, and oil/aqueous ratio of the mixture) and the hydrolysis rate.

R^2^ coefficients of the models are, respectively, 85.22%, 85.72%, and 82.36% for sunflower oil, hempseed oil, and linseed oil with CRL (Table 5).

The adjusted R^2^ shown in Table 5 indicates that more than 76.48% of the variability in the response could be explained by the model. These values of R^2^ reveal a satisfactory adjustment of the models to the experimental data, so these models can be used for the RSM analysis and the prediction of the hydrolysis rate.

An analysis of variance (ANOVA) was carried out to assess the significance of the fit of the response surface model for the hydrolysis rate. A *p*-value <0.05 is considered to be significant. The models are shown in Table 6.

The RSM analysis shows that, for sunflower oil, linear terms D and E, quadratic terms D^2^ and E^2^, and the interaction term D × E are significant ((a) in Table 6). For hempseed oil, linear terms D and E, quadratic terms D^2^ and E^2^, and the interaction terms D × E and D × O show a significant influence on the hydrolysis rate ((b) in Table 6). For linseed oil, linear terms D, E, and O and the quadratic term O^2^ are statistically significant ((c) in Table 6).

**Figure 4:**
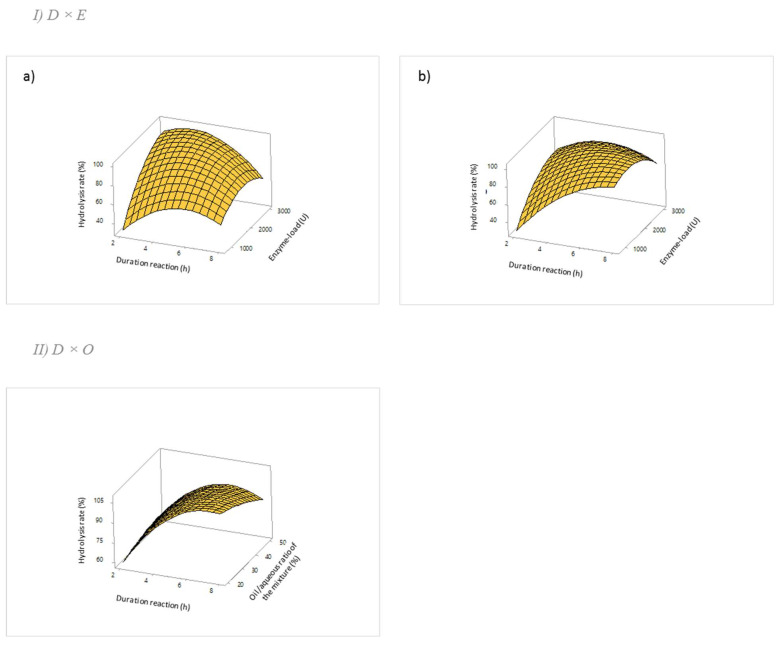
Three-dimensional (3D) response surface plots of the hydrolysis rate (%) with CRL, as a function of: (**I**) the duration reaction (D) and enzyme load (E) for sunflower oil (**a**) and hempseed oil (**b**); (**II**) the duration reaction (D) and oil/aqueous ratio of the mixture (O) for hempseed oil.

The effects of cross-term product factors on the rate of hydrolysis can be examined using three-dimensional (3D) response surface plots by maintaining a variable at its middle value. Only significant interactions are retained (Figure 4). The other 3D response surface plots are shown in the Supplementary Data, Figure S3.

On each plot, the third factor is maintained at its middle level. The holding values are 5 h for the duration reaction, 1900 U for the enzyme load, and 35% for the oil/aqueous ratio of the mixture.

Concerning the interaction term D × E, for a duration of 2 h, the larger the amount of enzyme, the higher the hydrolysis rate increases, rising from 40% with 1000 U of enzyme to over 80% with 3000 U, regardless of the oil selected (Figure 4I). For sunflower oil, a decrease of the hydrolysis rate can be observed after 4 h, regardless of the enzyme load. Although less pronounced, this decrease is still present for hempseed oil at a duration of 8 h for a high enzyme load (>2000 U). During the hydrolysis process, NaOH is continuously added to the medium, which could lead to the formation of soap when hydrolysis times are long. In this case, when FAs are saponified, they will not be quantified by the FTIR method, which would explain the lower hydrolysis rates measured.

The only variable between the two reactions being the lipid substrate, this would suggest that saponified FAs are formed more rapidly during the hydrolysis of sunflower oil than during the hydrolysis of hempseed oil. Indeed, sunflower oil contains high levels of PUFAs (over 55% linoleic acid) and also contains a high amount of monounsaturated and saturated FAs (around 43%), while hempseed oil contains more PUFAs (75%) and only 17% monounsaturated and saturated FAs (Table 1). Thus, the fatty acid composition would influence soap formation in the medium.

Concerning the interaction term D × O for hempseed oil, increasing the duration maximizes the hydrolysis rate, while the influence of the oil/aqueous ratio of the mixture remains low (Figure 4II). Indeed, the data previously show that D had a significant influence on the reaction, unlike O (Table 6).

The RSM approach provides optimal values of factors (duration reaction, enzyme load, and oil/aqueous ratio of the mixture) to optimize the reaction while maintaining a high hydrolysis rate. By maintaining a high hydrolysis rate, this optimization consisted of maximizing the amount of oil hydrolyzed in a single reaction while minimizing the reaction time and the amount of enzyme load. The RSM predicts the hydrolysis rates that could be reached in these optimal conditions. The predicted hydrolysis rate is then subject to experimental validation. The results are shown in Table 7.

For sunflower oil, a hydrolysis rate of 96.0 ± 1.7% is measured, i.e., with 1798 U of CRL at a pH of 7 and 35 °C over 4 h, 19.6 g of oil is hydrolyzed. The standard deviation is 0.2% with the predicted hydrolysis rate. This optimization allows 1.9-fold more oil to be hydrolyzed with 1.2-fold more enzyme over 4 h while maintaining a hydrolysis rate of 96% (Table 7, sunflower oil).

For hempseed oil, the maximum hydrolysis rate measured is 97.2 ± 3.8%, and the standard deviation with the predicted hydrolysis rate is 0.4%. With 2592 U of enzyme, 21.04 g of hempseed oil can be hydrolyzed in 4.5 h, which represents 2.1-fold more oil hydrolyzed with 1.9-fold more enzyme over 4.5 h compared with non-optimized reaction conditions (Table 7, hempseed oil).

Finally, a hydrolysis rate of linseed oil of 91.8 ± 3.2%% is found when the reaction is carried out under optimized conditions, i.e., in 4.5 h, with 1431 U of enzyme and an oil/water ratio of 38%, representing 15.34 g of oil hydrolyzed in one reaction. Under these conditions, 1.5-fold more oil can be hydrolyzed with 1.02-fold more enzyme over a period of 4.5 h, maintaining a high hydrolysis rate (91.8%). The standard deviation is 5.5% with the predicted hydrolysis rates (Table 7, linseed oil).

## 3. Materials and Methods

### 3.1. Oils and the Determination of Their Composition in Fatty Acids

Three commercial vegetable oils (hempseed oil, linseed oil, and sunflower oil) were chosen based on their high PUFA content. All are refined edible oils. Used in a biocatalytic process, an oil with a high linoleic acid content, such as sunflower oil, will allow, after the action of a lipase, a LOX, and an HPL, the synthesis of GLVs such as hexanal or (*Z*)-nonenal. On the other hand, an oil with a high linolenic acid content, such as linseed oil, will be used to obtain GLVs, like (*Z*,*Z*)-nona-3,6-dienal or (*Z*)-hex-3-enal. The choice of vegetable oil will therefore depend on the GLVs of interest that are desired.

In order to determine the composition and the fatty acid (FA) content of each of these selected oils, a transesterification was carried out according to the method described by Morrison and Smith (1964) [33]. Fatty acid methyl esters were recovered in 10 volumes of toluene (*w*/*v*).

FAs were identified by mass spectrometry (PerkinElmer SQ8C coupled to PerkinElmer Clarus 680) and quantified by gas chromatography with a flame ionization detector (PerkinElmer Clarus 600). The GC-FID was equipped with 60 m × 0.25 mm columns (film thickness 0.25 µm) and a nonpolar Rtx-1 (poly-dimethylsiloxane) phase. GC conditions were as follows: program temperature rose from 60 to 230 °C at 2 °C/min and was maintained at 230 °C for 30 min; injection temperature at 280 °C; detector temperature at 250 °C; carrier gas: H_2_ (0.7 mL/min); and split ratio: 1:50. The injected volume was 2 µL in automatic mode. The GC-MS quadrupole detector was equipped with the same column as described above. The analyses were conducted on a nonpolar column, and for each sample, reconstructed ionic chromatograms were collected and analyzed. GC conditions were the same as above, and MS conditions were as follows: ionization energy: 70 eV; ion source temperature: 150 °C; and EI-MS spectra obtained over a mass range of 35–350 amu during a scan time of 1 s. The injection volume for the sample was 2 µL in automatic mode.

### 3.2. Lipases and the Determination of Their Optimum Temperature and pH

The non-selective lipase *C. rugosa* (CRL) [34] and the 1,3-selective lipases from *R. miehei* (RML) and *P. fluorescens* (PFL-Amano) [24,25] were purchased at Sigma-Aldrich/Merck (Darmstadt, Germany).

For each enzyme (CRL, RML, and PFL), the optimum pH and optimum temperature were determined with a pH-STAT (877 Titrino plus, Metrohm) by monitoring the release of FAs from tributyrin. The determination of the enzyme activity was carried out for 5 min by titration with 0.05 N NaOH, which was added to keep the pH constant. The enzyme activity is expressed in enzyme units (U), and 1 U corresponds to 1 μmol of FA released per minute.

### 3.3. Optimization of the Lipase-Catalyzed Hydrolysis of Vegetable Oils

The three selected lipases as biocatalysts (CRL, RML, and PFL) were screened on the three selected oils (sunflower, hempseed, and linseed oil) in order to optimize the lipolysis reaction. An RSM was carried out to evaluate the influence of different factors (pH, temperature, duration reaction, enzyme load, and oil/aqueous ratio of the reaction mixture) on the hydrolysis rate. All hydrolysis reactions were monitored by a bioreactor (Lambda Minifor Laboratory fermenter) to control the reaction parameters.

First, the classic factors (pH and temperature) [35], which have a direct influence on the hydrolysis rate, were screened, and the results were analyzed statistically with a central composite design. The optimum pH and the optimum temperature of the reaction were determined for each oil/lipase combination, and for each oil, the lipase with the highest hydrolysis rate was selected for further experiments. In a second phase, the screening of the other factors of the reaction was carried out, and the data were statistically analyzed using a Box–Behnken design.

#### 3.3.1. Optimization of the Hydrolysis Rate Using a Central Composite Design

For each of the oils (sunflower, hempseed, and linseed oils) and lipases (CRL, RML, and PFL), a two-factor central composite design was used to study the combined effect of temperature and pH on the hydrolysis ratio. The central composite allows the use of RSM when less than 3 factors are investigated and offers the advantage of testing extreme values outside the limits of the experimental design. Preliminary experiments allowed for defining the limit values of the design. Table 8 provides information on the factor levels coded −1, 0, and 1 for low, middle, and high values, respectively.

For each hydrolysis, the response surface design was generated using Minitab Software (Release 18) and consisted of 39 experiments, including 3 repetitions. All experiments were led in a randomized order. The experimental designs with response values are shown in the Supplementary Materials, Table S1.

The other factors of the reaction (reaction duration, enzyme load, and oil/aqueous ratio) were set at levels defined by preliminary analyses. The reaction mixture (total volume of 50 mL) was prepared as follows: 14.3 g of vegetable oil (i.e., 24.5% (*v*/*v*)) was emulsified in the Ultraturrax (Ika T25) for 30 s at 13,500 rpm in a 150 mM NaCl solution containing 0.33% gum arabic. The mixture was then poured into a 0.3 L bioreactor vessel under agitation (8 Hz) to maintain the emulsion. The reaction was carried out at the temperature defined by the experimental design. The reaction started with the addition of 1390 U of enzyme and lasted 4 h. In order to maintain a constant pH, as defined in the design, despite the release of FAs, 0.05 N NaOH was added continuously to the bioreactor tank.

The RSM was applied to study the influence of the factors on the response. The optimum levels of pH and temperature were identified to obtain the combination values that maximize the hydrolysis rate. The lipase with the highest hydrolysis rate under optimal conditions was selected for further experimentation.

#### 3.3.2. Optimization of the Hydrolysis Reaction Using a Box–Behnken Design

The lipolysis optimization was studied next by investigating the influence of other reaction factors on the hydrolysis rate. An experimental design was carried out using an RSM Box–Behnken model in order to test the level of influence on the response of the reaction duration, enzyme load, and oil/aqueous ratio of the mixture. Preliminary experiments allowed for defining the limit values of the design. Table 9 provides information on the factor levels coded −1, 0, and 1 for low, middle, and high values, respectively.

For each oil, the response surface design consisted of 45 experiments, including 3 repetitions. All experiments were led in a randomized order. The experimental design with response values are shown in the Supplementary Materials, Table S2.

The reaction mixture (total volume of 50 mL) was prepared as follows: the volume of oil for each condition was emulsified at the ultraturax for 30 s at 13,500 rpm in a 150 mM NaCl solution containing 0.33% gum arabic. The mixture was then poured into a 0.3 L bioreactor vessel (Lambda Minifor Laboratory fermenter) under agitation (8 Hz) to maintain the emulsion. Hydrolysis was carried out at the optimum temperature and reaction pH previously defined for each oil. The pH was kept constant by adding 0.05 N NaOH. The reaction started by adding the amount of enzyme defined by the tested design condition. The reaction lasted for the duration defined by the design.

The data were analyzed statistically, and the best levels of factors were identified to provide prediction conditions that optimize the hydrolysis reaction. These conditions were then tested experimentally.

#### 3.3.3. Statistical Analysis of the Different Experimental Designs

The statistical analyses were performed using the response surface analysis module of the Minitab software. Regression analyses were carried out to fit the mathematical model to the experimental data obtained. The following generalized second-order polynomial equation describes the influence of the several factors tested on the response and was used to predict the optimal values:

$$\hat{Y}=\beta_{0}+\sum_{i=1}^{I}\beta_{i}X_{i}+\sum_{i=1}^{I}\beta_{ii}X_{i}^{2}+\sum_{i}\sum_{j}\beta_{ij}X_{i}X_{j}$$

where $\hat{Y}$ is the predicted response, *β*_0_ is the model constant, I is the number of factors (two in the central composite model and three in the Box–Behnken model), *β_i_* is the linear coefficient associated with factor *X_i_*, *β_ii_* is the quadratic coefficient associated with factor *X_i_*, and *β_ij_* is the interaction coefficient between factors *X_i_* and *X_j_*. In order to facilitate the reading and understanding of the results, factors *X_i_* were coded as follows: T for the temperature, H for the pH, D for the reaction duration, E for the enzyme load, and O for the oil/aqueous ratio.

An analysis of variance (ANOVA) was carried out, and the estimates of each coefficient as well as their significance level were determined. The adequacy of models was checked by determination coefficient (R^2^) analysis. The *p*-value was checked to find out the significance of all the fitted equation terms at a 5% level of significance.

The response surface plots of the predicted model were obtained by depicting two significant factors within the experimental range and keeping the other factors at their central level.

### 3.4. Lipids Analysis and Hydrolysis Rate Calculation

The saponification value for each oil was determined using the AOCS Cd3-25 method [36] in order to define the total amount of fatty acid in the oil. The saponification value, expressed in mgKOH.g^−1^ _of oil_ (*SV*_0_), was then converted to express the total fatty acid content in mol.g^−1^ _of oil_ (*SV*_0_′):

(1) $${SV}_{0}\prime =\frac{{SV}_{0}\times 10^{-3}}{{MW}_{KOH}}$$

where *SV*_0_′ is the total number of moles of FAs per gram of oil (mol.g^−1^_of oil_), *SV*_0_ is the saponification value of the oil (mgKOH.g^−1^_of oil_), and *MW_KOH_* is the molar weight of KOH (g.mol^−1^).

After each lipolysis reaction, the lipids of the reaction media were extracted according to the method of Folch et al. (1957) [37]. The released FAs were quantified by Fourier transform infrared (FTIR) spectroscopy (Alpha II, Bruker) using a calibration curve constructed from linoleic acid solutions at concentrations of 0–400 mM. Thin-layer chromatography analysis was performed to visualize triglycerides, diglycerides, monoglycerides, and free fatty acids (FFAs) in the medium. The hydrolysis rate was calculated from the saponification value of the oil as follows:

(2) $$Hydrolysis\;rate\;(\%)=\frac{n_{s}}{m_{o}\times {SV}_{0}\prime }\times 100$$

The ratio *n_s_/m_o_* corresponds to the number of moles of FAs released after the lipolysis per gram of oil (mol.g^−1^ _of oil_), where *n_s_* is the number of moles of FAs after the lipolysis (mol) and *m_o_* is the weight of oil in the reaction (g).

## 4. Conclusions

In this study, the optimization of the first step of the biocatalytic process for GLVs production, i.e., lipase-catalyzed hydrolysis, was investigated. We tested different suitable lipases and vegetable oils and several hydrolysis conditions (by varying factors including pH, temperature, duration reaction, enzyme load, and oil/aqueous ratio of the mixture). In order to optimize the hydrolysis reaction while maintaining high hydrolysis rates, experimental designs and RSM were used to determine optimal hydrolysis conditions.

The best hydrolysis rates were obtained using the CRL on the three vegetable oils selected (sunflower oil, hempseed oil, and linseed oil). Moreover, by varying reaction conditions, we were able to optimize the hydrolysis reaction by increasing the amount of oil hydrolyzed in a single reaction while maintaining very high hydrolysis rates (between 91.8% and 97.2%) and reaction times of 4 or 4.5 h. To conclude, the reaction has been optimized for the three oils, which can be used according to their lipid profile.

Finally, the optimized lipolysis reaction can be used in a vegetable oils bioconversion process for the biocatalytic production of GLVs.

## Figures and Tables

**Table 1:** Fatty acid composition of sunflower oil, hempseed oil, and linseed oil after analysis by chromatography with flame ionization detector and mass spectrometry.

	Content (%)		
	Sunflower Oil	Hempseed Oil	Linseed Oil
**Palmitic acid**	6.4 ± 0.0	6.1 ± 0.0	5.7 ± 0.1
**Stearic acid**	3.1 ± 0.0	6.6 ± 0.4	3.8 ± 0.0
**Oleic acid**	33.3 ± 0.1	4.4 ± 0.4	20.6 ± 0.1
**Linoleic acid**	55.3 ± 0.1	56.6 ± 0.1	15.5 ± 0.1
**Linolenic acid**	0.4 ± 0.0	19.0 ± 0.2	53.0 ± 0.1
**Others**	1.5 ± 0.0	7.3 ± 0.2	1.5 ± 0.1

**Table 2:** Response surface model analysis of the hydrolysis rate for the action of CRL, RML, and PFL on sunflower, hempseed, and linseed oils; second-order polynomial equations and determination coefficients.

Oil	Lipase	Equation ^a^	R^2^	Adjusted R^2^
**Sunflower oil**	**CRL**	Hydrolysis rate (%) = −1599.2 + 49.40 × T + 241.9 × H − 0.5417 × T^2^ − 13.347 × H^2^ − 1.672 × T × H	96.21%	95.60%
	**RML**	Hydrolysis rate (%) = −403.7 + 94.15 × H + 6.44 × T − 6.490 × H^2^ − 0.0813 × T^2^ − 0.098 × H × T	88.43%	86.68%
	**PFL**	Hydrolysis rate (%) = 75.65 − 17.52 × H + 0.445 × T + 0.8997 × H^2^ − 0.00894 × T^2^ + 0.0311 × H × T	89.83%	88.29%
**Hempseed oil**	**CRL**	Hydrolysis rate (%) = −30.26 + 6.419 × H + 0.6613 × T − 0.4771 × H^2^ − 0.00998 × T^2^ + 0.00557 × H × T	93.88%	92.95%
	**RML**	Hydrolysis rate (%) = −163.4 + 48.78 × H + 0.57 × T − 4.607 × H^2^ − 0.0698 × T^2^ + 0.5861 × H × T	89.94%	88.42%
	**PFL**	Hydrolysis rate (%) = −127.0 + 37.82 × H + 2.079 × T − 2.350 × H^2^ − 0.01762 × T^2^ − 0.0891 × H × T	91.99%	90.77%
**Linseed oil**	**CRL**	Hydrolysis rate (%) = −109 + 26.2 × H + 9.76 × T − 3.42 × H^2^ − 0.1808 × T + 0.176 × H × T	92.90%	90.29%
	**RML**	Hydrolysis rate (%) = −156.0 + 47.19 × H + 2.31 × T − 3.067 × H^2^ − 0.0051 × T − 0.2735 × H × T	91.20%	89.87%
	**PFL**	Hydrolysis rate (%) = 225.9 − 22.78 × H − 5.595 × T + 0.732 × H^2^ + 0.06655 × T^2^ + 0.1440 × H × T	92.42%	91.27%

^a^ Factors coded as follows: pH (H) and temperature (T).

**Table 3:** Analysis of variance (ANOVA): response surface model analysis of the hydrolysis rate with CRL, RML, and PFL.

(a) On Sunflower Oil												
Source ^a^	Sum of Squares			Degree of Freedom			*F* Value			*p*-Value ^b^		
	CRL	RML	PFL	CRL	RML	PFL	CRL	RML	PFL	CRL	RML	PFL
**H**	1958.80	2196.84	395.84	1	1	1	141.47	78.53	159.93	0.000	0.000	0.000
**T**	2795.60	242.73	10.95	1	1	1	60.44	8.68	4.42	0.000	0.006	0.043
**H^2^**	4124.70	4449.73	270.28	1	1	1	297.91	159.06	109.20	0.000	0.000	0.000
**T^2^**	4246.10	436.43	16.69	1	1	1	306.68	15.60	6.74	0.000	0.000	0.014
**H × T**	968.00	14.60	4.65	1	1	1	69.92	0.52	1.88	0.000	0.475	0.18
**Lack of fit**	413.10	607.59	39.21	3	3	3	238.58	19.25	9.23	0.000	0.000	0.000
**Pure error**	16.20	315.61	42.47	30	30	30						
**Total**	11,319.50	7978.28	802.89	38	38	38						
**(b) On Hempseed Oil**												
**Source ^a^**	**Sum of Squares**			**Degree of Freedom**			***F* Value**			***p*-Value ^b^**		
	**CRL**	**RML**	**PFL**	**CRL**	**RML**	**PFL**	**CRL**	**RML**	**PFL**	**CRL**	**RML**	**PFL**
**H**	16.67	87.66	805.85	1	1	1	173.73	7.99	111.58	0.000	0.008	0.000
**T**	4.034	246.45	42.1	1	1	1	42.05	22.46	5.83	0.000	0.000	0.021
**H^2^**	24.05	2242.35	1844.29	1	1	1	250.65	204.34	255.36	0.000	0.000	0.000
**T^2^**	6.57	321.40	64.83	1	1	1	68.50	29.29	8.98	0.000	0.000	0.005
**H × T**	0.05	521.69	38.1	1	1	1	0.49	47.54	5.28	0.488	0.000	0.028
**Lack of fit**	1.97	43.84	118.93	3	3	3	16.53	1.38	9.96	0.000	0.269	0.000
**Pure error**	1.19	318.28	119.4	30	30	30						
**Total**	51.72	3600.74	2974.79	38	38	38						
**(c) On Linseed Oil**												
**Source ^a^**	**Sum of Squares**			**Degree of Freedom**			***F* Value**			***p*-Value ^b^**		
	**CRL**	**RML**	**PFL**	**CRL**	**RML**	**PFL**	**CRL**	**RML**	**PFL**	**CRL**	**RML**	**PFL**
**H**	20.77	3204.88	3494.26	1	1	1	228.40	253.33	297.82	0.000	0.000	0.000
**T**	617.30	6.66	111.68	1	1	1	6.89	0.53	9.52	0.017	0.473	0.004
**H^2^**	437.90	993.60	178.83	1	1	1	4.88	78.54	15.24	0.04	0.000	0.000
**T^2^**	763.40	1.69	924.37	1	1	1	8.52	0.13	78.78	0.009	0.717	0.000
**H × T**	46.80	113.61	99.58	1	1	1	0.52	8.98	8.49	0.479	0.005	0.006
**Lack of fit**	1555.10	208.57	203.98	3	3	3	1.23	9.98	11.13	0.539	0.000	0
**Pure error**	148.30	208.91	183.21	30	30	30						
**Total**	3589.60	4744.26	5107.12	38	38	38						

^a^ Factors coded as follows: pH (H) and temperature (T); ^b^ *p*-value <0.05 indicates a statistical significance of the factor.

**Table 4:** Predicted and measured hydrolysis rates with optimal pH and temperature for the actions of CRL, RML, and PFL on sunflower, hempseed, and linseed oils.

	Sunflower Oil			Hempseed Oil			Linseed Oil		
	CRL	RML	PFL	CRL	RML	PFL	CRL	RML	PFL
**Optimal pH**	7	7	5.2	7.5	7.6	7.5	6	6	5.2
**Optimal temperature**	35 °C	35 °C	34 °C	35 °C	36 °C	35 °C	30 °C	43 °C	21 °C
**Predicted hydrolysis rate (%)**	96.2	39.8	19.7	86.7	31.5	52.8	90.6	36.3	55.4
**Measured hydrolysis rate (%)**	96.4 ± 1.8	38.4 ± 2.3	20.2 ± 1.5	87.9 ± 3.6	40.3 ± 3.2	48.7 ± 1.9	93.0 ± 3.8	39.0 ± 1.5	56.1 ± 2.7

**Table 5:** Response surface model analysis of the hydrolysis rate for the action of CRL on sunflower, hempseed, and linseed oils; second-order polynomial equations and determination coefficients.

Oil	Equation	R^2^	Adjusted R^2^
**Sunflower Oil**	Hydrolysis rate (%) = −6.234 + 1.270 × D + 0.003558 × E + 0.1333 × O − 0.0459 × D^2^ − 0.000000 × E^2^ − 0.001375 × O^2^ − 0.000270 × D × E − 0.00652 × D × O − 0.000002 × E × O	85.22%	81.43%
**Hempseed Oil**	Hydrolysis rate (%) = −87.9 + 37.50 × D + 0.0648 × E + 0.804 × O − 1.698 × D^2^ − 0.000011 × E^2^ − 0.0094 × O^2^ − 0.004911 × D × E + 0.000223 × E × O − 0.1567 × D × O	85.72%	82.05%
**Linseed Oil**	Hydrolysis rate (%) = −27.7 + 1.06 × D − 0.00701 × E + 6.446 × O + 0.213 × D^2^ + 0.00000 × E − 0.08542 × O^2^ − 0.000728 × D × E - 0.0146 × D × O + 0.000086 × E × O	82.36%	76.48%

**Table 6:** Response surface model analysis of the hydrolysis rate: analysis of variance (ANOVA).

(a) On Sunflower Oil				
Source ^a^	Sum of Squares	Degree of Freedom	*F* Value	*p*-Value ^b^
**D**	0.76	1	6.33	0.017
**E**	5.14	1	42.63	0.000
**O**	0.01	1	0.04	0.838
**D^2^**	7.42	1	61.46	0.000
**E^2^**	2.44	1	20.02	0.000
**O^2^**	0.31	1	2.57	0.118
**D × E**	2.05	1	16.99	0.000
**D × O**	0.17	1	1.39	0.246
**E × O**	0.34	1	2.85	0.1
**Lack of fit**	1.87	27	8.49	0.000
**Pure error**	2.36	32		
**Total**	22.01	44		
**(b) On Hempseed Oil**				
**Source ^a^**	**Sum of Squares**	**Degree of Freedom**	***F* Value**	***p*-Value ^b^**
**D**	7028.95	1	89.82	0.000
**E**	994.50	1	12.71	0.001
**O**	247.69	1	3.17	0.084
**D^2^**	2585.47	1	33.04	0.000
**E^2^**	2001.47	1	25.58	0.000
**O^2^**	49.48	1	0.63	0.432
**D × E**	3152.3	1	40.28	0.000
**D × O**	597.0	1	7.63	0.009
**E × O**	161.8	1	2.07	0.159
**Lack of fit**	1797.8	3	20.37	0.000
**Pure error**	941.3	32		
**Total**	19,183.4	44		
**(c) On Linseed Oil**				
**Source ^a^**	**Sum of Squares**	**Degree of Freedom**	***F* Value**	***p*-Value ^b^**
**D**	363.65	1	7.16	0.012
**E**	1425.89	1	28.06	0.000
**O**	1673.66	1	32.93	0.000
**D^2^**	40.66	1	0.8	0.378
**E^2^**	0.45	1	0.01	0.926
**O^2^**	4092.07	1	80.52	0.000
**D × E**	69.27	1	1.36	0.251
**D × O**	5.18	1	0.1	0.752
**E × O**	24.12	1	0.47	0.496
**Lack of fit**	1664.57	27	29.48	0.000
**Pure error**	12.55	6		
**Total**	9509.25	44		

^a^ Factors coded as follows: duration reaction (D), enzyme load (E), and oil/aqueous ratio of the mixture (O); ^b^ *p*-value < 0.05 indicates a statistical significance of the factor.

**Table 7:** Predicted and measured hydrolysis rates with optimal conditions of duration reaction, enzyme load, oil/aqueous ratio of the mixture, pH, and temperature for the action of CRL on sunflower, hempseed, and linseed oils.

Oils	CRL		
	Optimum Conditions of Reaction	Predicted Hydrolysis Rate (%)	Measured Hydrolysis Rate (%)
**Sunflower oil**	4 h − 1 798 U − 46.5% (oil/aqueous) 35 °C − pH 7	96.2	96.0 ± 1.7
**Hempseed oil**	4.5 h − 2 592 U − 49.2% (oil/aqueous) 35 °C − pH 7.5	97.6	97.2 ± 3.8
**Linseed oil**	4.5 h − 1 431 U − 38% (oil/aqueous) 30 °C − pH 6	96.9	91.8 ± 3.2

**Table 8:** Experimental levels of factors used in the trials for each lipase and each oil.

Factor	Lipases					
	CRL ^a^, RML ^a^			PFL ^a^		
	Coded Factor Levels			Coded Factor Levels		
	−1 (Low)	0 (Middle)	+1 (High)	−1 (Low)	0 (Middle)	+1 (High)
**pH**	6	7.5	9	6	8	10
**Temperature (°C)**	25	32.5	40	25	35	45

^a^ CRL: Candida rugosa lipase; RML: Rhizomucor miehei lipase; PFL: Pseudomonas fluorescens lipase.

**Table 9:** Experimental levels of factors used in the trials for each oil.

Factors	Coded Factor Levels		
	−1 (Low)	0 (Middle)	+1 (High)
Reaction duration (h)	2	5	8
Enzyme load (U)	800	1900	3000
Oil/aqueous ratio of the mixture (%)	10	35	50

## Supplementary Materials

The following supporting information can be downloaded at: https://www.mdpi.com/article/10.3390/ijms241512274/s1.
