# Supplementary material for: Optimizing the First Step of the Biocatalytic Process for Green Leaf Volatiles Production: Lipase-Catalyzed Hydrolysis of Three Vegetable Oils
Source: Int J Mol Sci. 2023 Jul 31;24(15):12274. doi: 10.3390/ijms241512274 (PMC10418742; doi:10.3390/ijms241512274)
Supplement: Supplementary file 1 [file ijms-24-12274-s001.zip › ijms-2525601-Supplementary Materials.pdf]

# Supplementary Materials

**Table S1.** Experimental design (Central composite) for each oil and each lipase

**a) Sunflower oil**

1) CRL

| Sample number (Standard Order) | Randomized Order | pH | Temperature (°C) | Hydrolysis rate (%) |
|---|---|---|---|---|
| 1 | 34 | 9.6 | 32.5 | 6.1 |
| 2 | 17 | 9.0 | 40.0 | 10.3 |
| 3 | 37 | 7.5 | 32.5 | 88.2 |
| 4 | 21 | 9.6 | 32.5 | 6.7 |
| 5 | 30 | 9.0 | 40.0 | 10.3 |
| 6 | 38 | 7.5 | 32.5 | 87.5 |
| 7 | 32 | 7.5 | 43.1 | 34.1 |
| 8 | 10 | 7.5 | 32.5 | 92.1 |
| 9 | 29 | 9.0 | 25.0 | 7.7 |
| 10 | 14 | 6.0 | 25.0 | 12.4 |
| 11 | 25 | 7.5 | 32.5 | 91.1 |
| 12 | 24 | 7.5 | 32.5 | 89.0 |
| 13 | 12 | 7.5 | 32.5 | 89.3 |
| 14 | 18 | 7.5 | 21.9 | 20.3 |
| 15 | 11 | 7.5 | 32.5 | 89.6 |
| 16 | 35 | 7.5 | 32.5 | 92.1 |
| 17 | 3 | 9.0 | 25.0 | 7.6 |
| 18 | 7 | 5.4 | 32.5 | 50.7 |
| 19 | 39 | 7.5 | 32.5 | 89.3 |
| 20 | 15 | 6.0 | 40.0 | 92.6 |
| 21 | 36 | 7.5 | 32.5 | 87.2 |
| 22 | 8 | 9.6 | 32.5 | 7.1 |
| 23 | 27 | 6.0 | 25.0 | 17.9 |
| 24 | 13 | 7.5 | 32.5 | 92.1 |
| 25 | 31 | 7.5 | 21.9 | 20.7 |
| 26 | 26 | 7.5 | 32.5 | 93.2 |
| 27 | 2 | 6.0 | 40.0 | 93.7 |
| 28 | 6 | 7.5 | 43.1 | 35.9 |
| 29 | 5 | 7.5 | 21.9 | 21.6 |
| 30 | 22 | 7.5 | 32.5 | 89.3 |
| 31 | 1 | 6.0 | 25.0 | 15.1 |
| 32 | 33 | 5.4 | 32.5 | 48.3 |
| 33 | 23 | 7.5 | 32.5 | 89.6 |
| 34 | 9 | 7.5 | 32.5 | 93.1 |
| 35 | 19 | 7.5 | 43.1 | 32.0 |
| 36 | 4 | 9.0 | 40.0 | 10.2 |
| 37 | 28 | 6.0 | 40.0 | 92.2 |
| 38 | 20 | 5.4 | 32.5 | 51.0 |
| 39 | 16 | 9.0 | 25.0 | 7.9 |

2) PFL

| Sample number (Standard Order) | Randomized Order | pH | Temperature (°C) | Hydrolysis rate (%) |
|---|---|---|---|---|
| 1 | 3 | 6.0 | 45.0 | 12.0 |
| 2 | 36 | 8.0 | 35.0 | 7.2 |
| 3 | 29 | 6.0 | 45.0 | 12.4 |
| 4 | 25 | 8.0 | 35.0 | 5.2 |
| 5 | 34 | 8.0 | 49.1 | 4.8 |
| 6 | 12 | 8.0 | 35.0 | 6.9 |
| 7 | 39 | 8.0 | 35.0 | 6.2 |
| 8 | 2 | 10.0 | 25.0 | 7.3 |
| 9 | 26 | 8.0 | 35.0 | 7.8 |
| 10 | 1 | 6.0 | 25.0 | 15.0 |
| 11 | 5 | 5.2 | 35.0 | 21.0 |
| 12 | 32 | 10.8 | 35.0 | 5.5 |
| 13 | 28 | 10.0 | 25.0 | 5.6 |
| 14 | 4 | 10.0 | 45.0 | 9.5 |
| 15 | 22 | 8.0 | 35.0 | 7.0 |
| 16 | 27 | 6.0 | 25.0 | 12.7 |
| 17 | 31 | 5.2 | 35.0 | 21.5 |
| 18 | 30 | 10.0 | 45.0 | 5.9 |
| 19 | 19 | 10.8 | 35.0 | 6.7 |
| 20 | 10 | 8.0 | 35.0 | 4.1 |
| 21 | 13 | 8.0 | 35.0 | 6.2 |
| 22 | 21 | 8.0 | 49.1 | 7.5 |
| 23 | 14 | 6.0 | 25.0 | 12.9 |
| 24 | 9 | 8.0 | 35.0 | 6.0 |
| 25 | 16 | 6.0 | 45.0 | 12.7 |
| 26 | 11 | 8.0 | 35.0 | 6.2 |
| 27 | 24 | 8.0 | 35.0 | 6.7 |
| 28 | 37 | 8.0 | 35.0 | 7.3 |
| 29 | 15 | 10.0 | 25.0 | 5.0 |
| 30 | 8 | 8.0 | 49.1 | 5.2 |
| 31 | 18 | 5.2 | 35.0 | 17.4 |
| 32 | 38 | 8.0 | 35.0 | 7.1 |
| 33 | 6 | 10.8 | 35.0 | 5.6 |
| 34 | 17 | 10.0 | 45.0 | 6.5 |
| 35 | 7 | 8.0 | 20.9 | 3.2 |
| 36 | 33 | 8.0 | 20.9 | 1.4 |
| 37 | 23 | 8.0 | 35.0 | 6.0 |
| 38 | 35 | 8.0 | 35.0 | 6.5 |
| 39 | 20 | 8.0 | 20.9 | 1.8 |

3) RML

| Sample number (Standard Order) | Randomized Order | pH | Temperature (°C) | Hydrolysis rate (%) |
|---|---|---|---|---|
| 1 | 28 | 9.0 | 25.0 | 1.5 |
| 2 | 10 | 7.5 | 32.5 | 39.2 |
| 3 | 8 | 7.5 | 43.1 | 36.3 |
| 4 | 20 | 7.5 | 21.9 | 24.0 |
| 5 | 9 | 7.5 | 32.5 | 37.5 |
| 6 | 36 | 7.5 | 32.5 | 37.2 |
| 7 | 14 | 6.0 | 25.0 | 23.2 |
| 8 | 37 | 7.5 | 32.5 | 34.2 |
| 9 | 11 | 7.5 | 32.5 | 31.2 |
| 10 | 39 | 7.5 | 32.5 | 30.7 |
| 11 | 12 | 7.5 | 32.5 | 41.3 |
| 12 | 33 | 7.5 | 21.9 | 27.8 |
| 13 | 31 | 5.4 | 32.5 | 22.4 |
| 14 | 7 | 7.5 | 21.9 | 21.6 |
| 15 | 38 | 7.5 | 32.5 | 38.7 |
| 16 | 1 | 6.0 | 25.0 | 24.6 |
| 17 | 22 | 7.5 | 32.5 | 35.4 |
| 18 | 27 | 6.0 | 25.0 | 23.9 |
| 19 | 3 | 6.0 | 40.0 | 28.2 |
| 20 | 15 | 9.0 | 25.0 | 1.8 |
| 21 | 32 | 9.6 | 32.5 | 1.8 |
| 22 | 21 | 7.5 | 43.1 | 32.6 |
| 23 | 5 | 5.4 | 32.5 | 18.3 |
| 24 | 17 | 9.0 | 40.0 | 1.9 |
| 25 | 6 | 9.6 | 32.5 | 1.7 |
| 26 | 13 | 7.5 | 32.5 | 36.0 |
| 27 | 29 | 6.0 | 40.0 | 28.3 |
| 28 | 18 | 5.4 | 32.5 | 23.7 |
| 29 | 35 | 7.5 | 32.5 | 37.0 |
| 30 | 30 | 9.0 | 40.0 | 2.0 |
| 31 | 23 | 7.5 | 32.5 | 40.0 |
| 32 | 4 | 9.0 | 40.0 | 2.0 |
| 33 | 19 | 9.6 | 32.5 | 1.9 |
| 34 | 16 | 6.0 | 40.0 | 29.2 |
| 35 | 24 | 7.5 | 32.5 | 36.0 |
| 36 | 26 | 7.5 | 32.5 | 39.0 |
| 37 | 2 | 9.0 | 25.0 | 1.8 |
| 38 | 25 | 7.5 | 32.5 | 42.0 |
| 39 | 34 | 7.5 | 43.1 | 48.0 |

**b. Hempseed oil**

1) CRL

| Sample number (Standard Order) | Randomized Order | pH | Temperature (°C) | Hydrolysis rate (%) |
|---|---|---|---|---|
| 1 | 24 | 7.5 | 32.5 | 96.3 |
| 2 | 32 | 9.6 | 32.5 | 11.5 |
| 3 | 20 | 7.5 | 21.9 | 47.7 |
| 4 | 39 | 7.5 | 32.5 | 94.3 |
| 5 | 21 | 7.5 | 43.1 | 92.0 |
| 6 | 9 | 7.5 | 32.5 | 94.0 |
| 7 | 6 | 9.6 | 32.5 | 12.3 |
| 8 | 28 | 9.0 | 25.0 | 14.9 |
| 9 | 27 | 6.0 | 25.0 | 75.9 |
| 10 | 4 | 9.0 | 40.0 | 32.1 |
| 11 | 29 | 6.0 | 40.0 | 79.7 |
| 12 | 11 | 7.5 | 32.5 | 94.2 |
| 13 | 13 | 7.5 | 32.5 | 92.4 |
| 14 | 8 | 7.5 | 43.1 | 89.4 |
| 15 | 19 | 9.6 | 32.5 | 0.0 |
| 16 | 16 | 6.0 | 40.0 | 80.5 |
| 17 | 15 | 9.0 | 25.0 | 15.3 |
| 18 | 22 | 7.5 | 32.5 | 100.0 |
| 19 | 2 | 9.0 | 25.0 | 11.3 |
| 20 | 35 | 7.5 | 32.5 | 95.4 |
| 21 | 5 | 5.4 | 32.5 | 66.1 |
| 22 | 31 | 5.4 | 32.5 | 63.3 |
| 23 | 34 | 7.5 | 43.1 | 90.8 |
| 24 | 7 | 7.5 | 21.9 | 48.1 |
| 25 | 25 | 7.5 | 32.5 | 99.7 |
| 26 | 17 | 9.0 | 40.0 | 26.6 |
| 27 | 37 | 7.5 | 32.5 | 96.6 |
| 28 | 26 | 7.5 | 32.5 | 93.1 |
| 29 | 1 | 6.0 | 25.0 | 74.4 |
| 30 | 10 | 7.5 | 32.5 | 100.0 |
| 31 | 36 | 7.5 | 32.5 | 97.4 |
| 32 | 3 | 6.0 | 40.0 | 81.9 |
| 33 | 23 | 7.5 | 32.5 | 94.3 |
| 34 | 33 | 7.5 | 21.9 | 19.1 |
| 35 | 14 | 6.0 | 25.0 | 72.9 |
| 36 | 18 | 5.4 | 32.5 | 57.8 |
| 37 | 30 | 9.0 | 40.0 | 23.2 |
| 38 | 12 | 7.5 | 32.5 | 92.1 |
| 39 | 38 | 7.5 | 32.5 | 95.7 |

2) PFL

| Sample number (Standard Order) | Randomized Order | pH | Temperature (°C) | Hydrolysis rate (%) |
|---|---|---|---|---|
| 1 | 16 | 6.0 | 45.0 | 44.2 |
| 2 | 12 | 8.0 | 35.0 | 51.9 |
| 3 | 22 | 8.0 | 35.0 | 48.8 |
| 4 | 9 | 8.0 | 35.0 | 54.4 |
| 5 | 35 | 8.0 | 35.0 | 52.4 |
| 6 | 30 | 10.0 | 45.0 | 32.5 |
| 7 | 27 | 6.0 | 25.0 | 46.2 |
| 8 | 20 | 8.0 | 20.9 | 42.3 |
| 9 | 2 | 10.0 | 25.0 | 40.1 |
| 10 | 21 | 8.0 | 49.1 | 50.8 |
| 11 | 37 | 8.0 | 35.0 | 49.8 |
| 12 | 10 | 8.0 | 35.0 | 51.9 |
| 13 | 5 | 5.2 | 35.0 | 44.2 |
| 14 | 17 | 10.0 | 45.0 | 36.1 |
| 15 | 4 | 10.0 | 45.0 | 33.0 |
| 16 | 28 | 10.0 | 25.0 | 38.1 |
| 17 | 15 | 10.0 | 25.0 | 37.3 |
| 18 | 33 | 8.0 | 20.9 | 42.7 |
| 19 | 39 | 8.0 | 35.0 | 50.3 |
| 20 | 34 | 8.0 | 49.1 | 52.3 |
| 21 | 7 | 8.0 | 20.9 | 42.2 |
| 22 | 29 | 6.0 | 45.0 | 51.2 |
| 23 | 26 | 8.0 | 35.0 | 50.8 |
| 24 | 13 | 8.0 | 35.0 | 52.4 |
| 25 | 18 | 5.2 | 35.0 | 36.6 |
| 26 | 36 | 8.0 | 35.0 | 49.3 |
| 27 | 32 | 10.8 | 35.0 | 21.4 |
| 28 | 14 | 6.0 | 25.0 | 45.2 |
| 29 | 19 | 10.8 | 35.0 | 22.4 |
| 30 | 6 | 10.8 | 35.0 | 23.4 |
| 31 | 1 | 6.0 | 25.0 | 43.7 |
| 32 | 24 | 8.0 | 35.0 | 48.8 |
| 33 | 3 | 6.0 | 45.0 | 47.3 |
| 34 | 38 | 8.0 | 35.0 | 51.3 |
| 35 | 31 | 5.2 | 35.0 | 41.7 |
| 36 | 23 | 8.0 | 35.0 | 52.4 |
| 37 | 25 | 8.0 | 35.0 | 51.9 |
| 38 | 8 | 8.0 | 49.1 | 50.9 |
| 39 | 11 | 8.0 | 35.0 | 54.9 |

3) RML

| Sample number (Standard Order) | Randomized Order | pH | Temperature (°C) | Hydrolysis rate (%) |
|---|---|---|---|---|
| 1 | 16 | 6.0 | 40.0 | 9.3 |
| 2 | 12 | 7.5 | 32.5 | 32.2 |
| 3 | 22 | 7.5 | 32.5 | 30.4 |
| 4 | 9 | 7.5 | 32.5 | 31.2 |
| 5 | 35 | 7.5 | 32.5 | 34.1 |
| 6 | 30 | 9.0 | 40.0 | 26.3 |
| 7 | 27 | 6.0 | 25.0 | 22.3 |
| 8 | 20 | 7.5 | 21.9 | 21.7 |
| 9 | 2 | 9.0 | 25.0 | 0.0 |
| 10 | 21 | 7.5 | 43.1 | 34.7 |
| 11 | 37 | 7.5 | 32.5 | 29.7 |
| 12 | 10 | 7.5 | 32.5 | 27.9 |
| 13 | 5 | 5.4 | 32.5 | 13.0 |
| 14 | 17 | 9.0 | 40.0 | 26.1 |
| 15 | 4 | 9.0 | 40.0 | 23.4 |
| 16 | 28 | 9.0 | 25.0 | 2.5 |
| 17 | 15 | 9.0 | 25.0 | 8.5 |
| 18 | 33 | 7.5 | 21.9 | 18.0 |
| 19 | 39 | 7.5 | 32.5 | 29.7 |
| 20 | 34 | 7.5 | 43.1 | 23.5 |
| 21 | 7 | 7.5 | 21.9 | 22.3 |
| 22 | 29 | 6.0 | 40.0 | 18.0 |
| 23 | 26 | 7.5 | 32.5 | 26.0 |
| 24 | 13 | 7.5 | 32.5 | 26.6 |
| 25 | 18 | 5.4 | 32.5 | 14.9 |
| 26 | 36 | 7.5 | 32.5 | 29.1 |
| 27 | 32 | 9.6 | 32.5 | 6.8 |
| 28 | 14 | 6.0 | 25.0 | 17.3 |
| 29 | 19 | 9.6 | 32.5 | 5.6 |
| 30 | 6 | 9.6 | 32.5 | 9.9 |
| 31 | 1 | 6.0 | 25.0 | 19.2 |
| 32 | 24 | 7.5 | 32.5 | 34.1 |
| 33 | 3 | 6.0 | 40.0 | 17.3 |
| 34 | 38 | 7.5 | 32.5 | 32.2 |
| 35 | 31 | 5.4 | 32.5 | 15.0 |
| 36 | 23 | 7.5 | 32.5 | 34.1 |
| 37 | 25 | 7.5 | 32.5 | 33.5 |
| 38 | 8 | 7.5 | 43.1 | 22.3 |
| 39 | 11 | 7.5 | 32.5 | 32.2 |

**c. Linseed oil**

1) CRL

| Sample number (Standard Order) | Randomized Order | pH | Temperature (°C) | Hydrolysis rate (%) |
|---|---|---|---|---|
| 1 | 6 | 9.6 | 32.5 | 9.4 |
| 2 | 12 | 7.5 | 32.5 | 57.9 |
| 3 | 10 | 7.5 | 32.5 | 62.7 |
| 4 | 1 | 6.0 | 25.0 | 70.6 |
| 5 | 5 | 5.4 | 32.5 | 100.0 |
| 6 | 7 | 7.5 | 21.9 | 58.4 |
| 7 | 11 | 7.5 | 32.5 | 74.6 |
| 8 | 13 | 7.5 | 32.5 | 58.6 |
| 9 | 4 | 9.0 | 40.0 | 9.1 |
| 10 | 3 | 6.0 | 40.0 | 58.0 |
| 11 | 9 | 7.5 | 32.5 | 55.7 |
| 12 | 8 | 7.5 | 43.1 | 60.7 |
| 13 | 2 | 9.0 | 25.0 | 11.3 |
| 14 | 19 | 9.6 | 32.5 | 12.8 |
| 15 | 25 | 7.5 | 32.5 | 56.2 |
| 16 | 23 | 7.5 | 32.5 | 57.8 |
| 17 | 14 | 6.0 | 25.0 | 72.1 |
| 18 | 18 | 5.4 | 32.5 | 89.7 |
| 19 | 20 | 7.5 | 21.9 | 56.0 |
| 20 | 24 | 7.5 | 32.5 | 56.7 |
| 21 | 26 | 7.5 | 32.5 | 58.4 |
| 22 | 17 | 9.0 | 40.0 | 10.1 |
| 23 | 16 | 6.0 | 40.0 | 64.7 |
| 24 | 22 | 7.5 | 32.5 | 58.1 |
| 25 | 21 | 7.5 | 43.1 | 29.9 |
| 26 | 15 | 9.0 | 25.0 | 14.8 |
| 27 | 32 | 9.6 | 32.5 | 8.3 |
| 28 | 38 | 7.5 | 32.5 | 58.9 |
| 29 | 36 | 7.5 | 32.5 | 57.7 |
| 30 | 27 | 6.0 | 25.0 | 78.8 |
| 31 | 31 | 5.4 | 32.5 | 98.0 |
| 32 | 33 | 7.5 | 21.9 | 57.2 |
| 33 | 37 | 7.5 | 32.5 | 57.5 |
| 34 | 39 | 7.5 | 32.5 | 59.1 |
| 35 | 30 | 9.0 | 40.0 | 11.4 |
| 36 | 29 | 6.0 | 40.0 | 64.7 |
| 37 | 35 | 7.5 | 32.5 | 57.0 |
| 38 | 34 | 7.5 | 43.1 | 26.4 |
| 39 | 28 | 9.0 | 25.0 | 14.9 |

2) PFL

| Sample number (Standard Order) | Randomized Order | pH | Temperature (°C) | Hydrolysis rate (%) |
|---|---|---|---|---|
| 1 | 16 | 6.0 | 45.0 | 32.0 |
| 2 | 12 | 8.0 | 35.0 | 14.2 |
| 3 | 22 | 8.0 | 35.0 | 19.3 |
| 4 | 9 | 8.0 | 35.0 | 16.8 |
| 5 | 35 | 8.0 | 35.0 | 17.3 |
| 6 | 30 | 10.0 | 45.0 | 14.7 |
| 7 | 27 | 6.0 | 25.0 | 37.1 |
| 8 | 20 | 8.0 | 20.9 | 29.0 |
| 9 | 2 | 10.0 | 25.0 | 10.2 |
| 10 | 21 | 8.0 | 49.1 | 34.6 |
| 11 | 37 | 8.0 | 35.0 | 16.3 |
| 12 | 10 | 8.0 | 35.0 | 17.3 |
| 13 | 5 | 5.2 | 35.0 | 43.7 |
| 14 | 17 | 10.0 | 45.0 | 23.4 |
| 15 | 4 | 10.0 | 45.0 | 22.9 |
| 16 | 28 | 10.0 | 25.0 | 9.2 |
| 17 | 15 | 10.0 | 25.0 | 9.7 |
| 18 | 33 | 8.0 | 20.9 | 29.5 |
| 19 | 39 | 8.0 | 35.0 | 14.7 |
| 20 | 34 | 8.0 | 49.1 | 31.0 |
| 21 | 7 | 8.0 | 20.9 | 27.5 |
| 22 | 29 | 6.0 | 45.0 | 30.0 |
| 23 | 26 | 8.0 | 35.0 | 15.3 |
| 24 | 13 | 8.0 | 35.0 | 14.2 |
| 25 | 18 | 5.2 | 35.0 | 46.3 |
| 26 | 36 | 8.0 | 35.0 | 16.8 |
| 27 | 32 | 10.8 | 35.0 | 3.6 |
| 28 | 14 | 6.0 | 25.0 | 30.5 |
| 29 | 19 | 10.8 | 35.0 | 2.5 |
| 30 | 6 | 10.8 | 35.0 | 3.6 |
| 31 | 1 | 6.0 | 25.0 | 36.1 |
| 32 | 24 | 8.0 | 35.0 | 18.8 |
| 33 | 3 | 6.0 | 45.0 | 39.1 |
| 34 | 38 | 8.0 | 35.0 | 18.3 |
| 35 | 31 | 5.2 | 35.0 | 43.2 |
| 36 | 23 | 8.0 | 35.0 | 13.7 |
| 37 | 25 | 8.0 | 35.0 | 17.8 |
| 38 | 8 | 8.0 | 49.1 | 36.1 |
| 39 | 11 | 8.0 | 35.0 | 16.3 |

3) RML

| Sample number (Standard Order) | Randomized Order | pH | Temperature (°C) | Hydrolysis rate (%) |
|---|---|---|---|---|
| 1 | 16 | 6.0 | 40.0 | 42.2 |
| 2 | 12 | 7.5 | 32.5 | 28.3 |
| 3 | 22 | 7.5 | 32.5 | 28.8 |
| 4 | 9 | 7.5 | 32.5 | 28.3 |
| 5 | 35 | 7.5 | 32.5 | 28.5 |
| 6 | 30 | 9.0 | 40.0 | 2.5 |
| 7 | 27 | 6.0 | 25.0 | 40.2 |
| 8 | 20 | 7.5 | 21.9 | 34.2 |
| 9 | 2 | 9.0 | 25.0 | 10.2 |
| 10 | 21 | 7.5 | 43.1 | 27.3 |
| 11 | 37 | 7.5 | 32.5 | 28.1 |
| 12 | 10 | 7.5 | 32.5 | 28.6 |
| 13 | 5 | 5.4 | 32.5 | 27.5 |
| 14 | 17 | 9.0 | 40.0 | 3.1 |
| 15 | 4 | 9.0 | 40.0 | 1.9 |
| 16 | 28 | 9.0 | 25.0 | 11.6 |
| 17 | 15 | 9.0 | 25.0 | 7.8 |
| 18 | 33 | 7.5 | 21.9 | 23.9 |
| 19 | 39 | 7.5 | 32.5 | 29.0 |
| 20 | 34 | 7.5 | 43.1 | 28.6 |
| 21 | 7 | 7.5 | 21.9 | 29.7 |
| 22 | 29 | 6.0 | 40.0 | 32.6 |
| 23 | 26 | 7.5 | 32.5 | 28.8 |
| 24 | 13 | 7.5 | 32.5 | 28.3 |
| 25 | 18 | 5.4 | 32.5 | 25.7 |
| 26 | 36 | 7.5 | 32.5 | 28.3 |
| 27 | 32 | 9.6 | 32.5 | 3.7 |
| 28 | 14 | 6.0 | 25.0 | 28.0 |
| 29 | 19 | 9.6 | 32.5 | 3.4 |
| 30 | 6 | 9.6 | 32.5 | 2.2 |
| 31 | 1 | 6.0 | 25.0 | 29.5 |
| 32 | 24 | 7.5 | 32.5 | 28.0 |
| 33 | 3 | 6.0 | 40.0 | 37.6 |
| 34 | 38 | 7.5 | 32.5 | 28.5 |
| 35 | 31 | 5.4 | 32.5 | 30.0 |
| 36 | 23 | 7.5 | 32.5 | 28.8 |
| 37 | 25 | 7.5 | 32.5 | 28.5 |
| 38 | 8 | 7.5 | 43.1 | 28.1 |
| 39 | 11 | 7.5 | 32.5 | 28.6 |

*a) Sunflower oil*

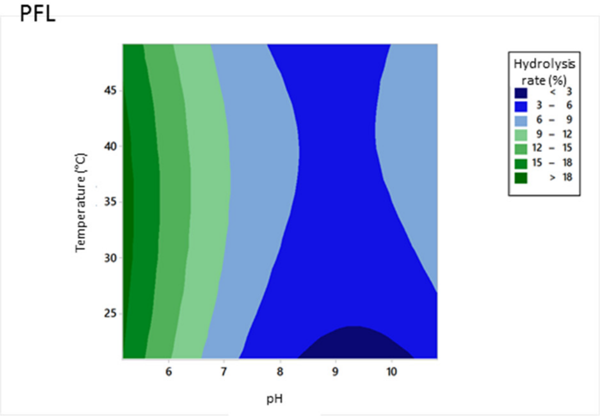


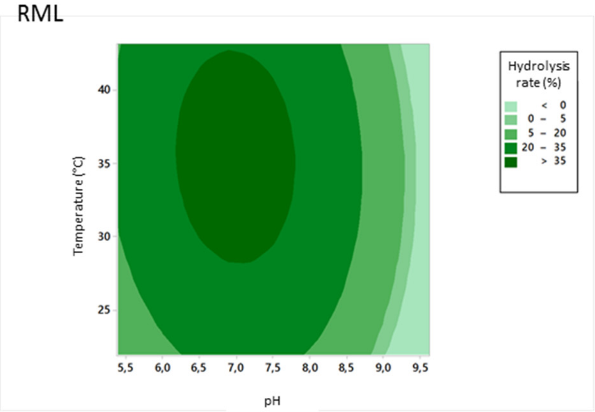


*b) Hempseed oil*

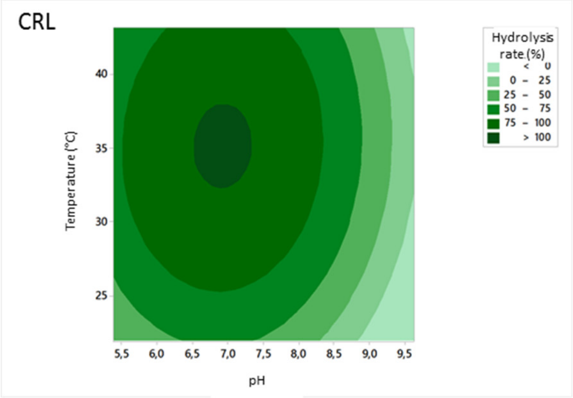


*c) Linseed oil*

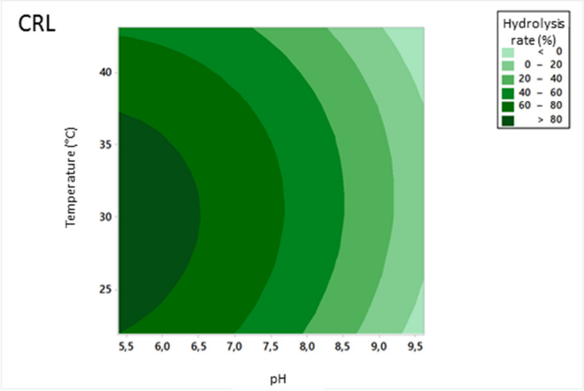


**Figure S1**. Contour plots of the hydrolysis rate (%) after lipolysis on sunflower oil (a) hempseed oil (b) and linseed oil (c), as a function of the temperature (T) and pH (H) for CRL, PFL and RML

*a) Sunflower oil*

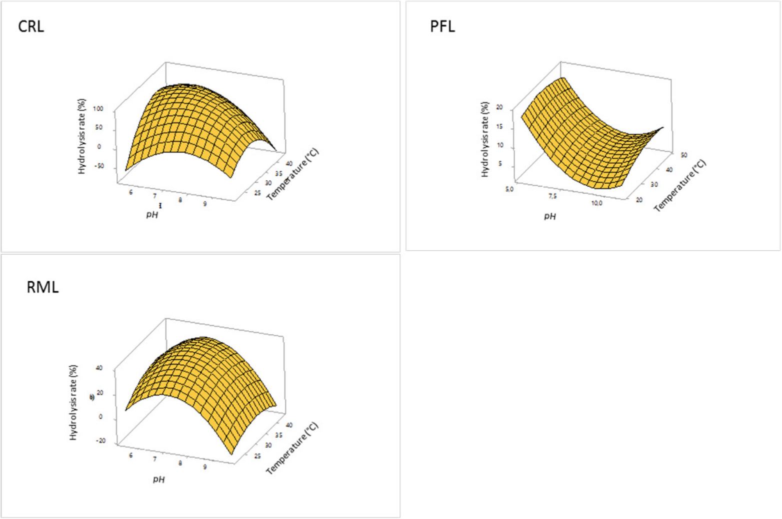


*b) Hempseed oil*

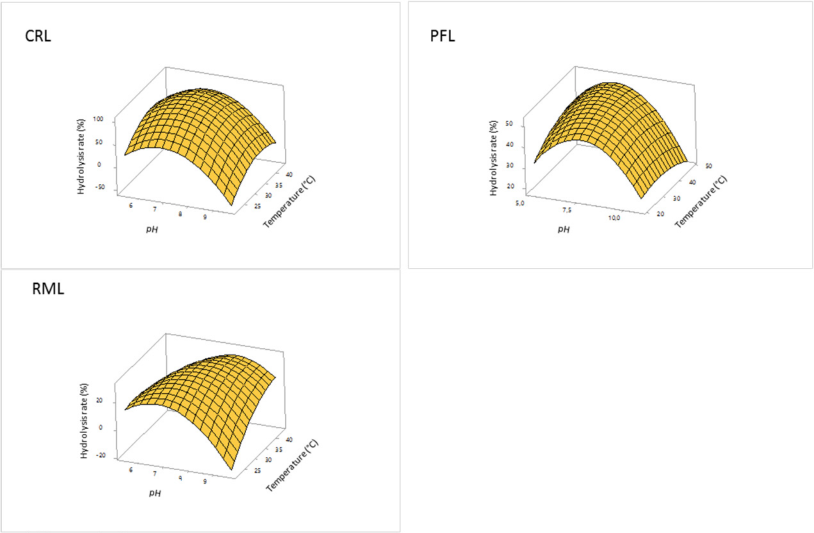


*c) Linseed oil*

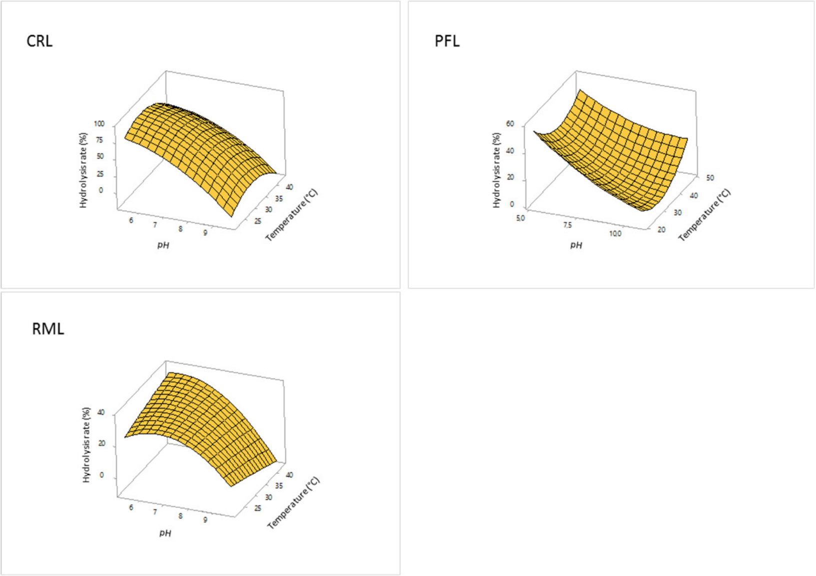


**Figure S2**. Three-dimensional (3D) response surface plots of the hydrolysis rate (%) after lipolysis on sunflower oil (a) hempseed oil (b) and linseed oil (c), as a function of the temperature (T) and pH (H) for CRL, PFL and RML

**Table S2**. Experimental design (Box-Behnken) for each oil with CRL

a. Sunflower oil

| Sample number (Standard Order) | Randomized Order | Duration reaction (h) | Enzyme load (U) | Oil/aqueous ratio of the mixture (%) | Hydrolysis rate (%) |
|---|---|---|---|---|---|
| 1 | 1 | 2 | 800 | 35 | 44.1 |
| 2 | 6 | 8 | 1900 | 20 | 62.5 |
| 3 | 7 | 2 | 1900 | 50 | 72.5 |
| 4 | 2 | 8 | 800 | 35 | 51.6 |
| 5 | 15 | 5 | 1900 | 35 | 93.7 |
| 6 | 45 | 5 | 1900 | 35 | 93.1 |
| 7 | 39 | 5 | 800 | 20 | 64.5 |
| 8 | 11 | 5 | 800 | 50 | 45.6 |
| 9 | 9 | 5 | 800 | 20 | 60.2 |
| 10 | 4 | 8 | 3000 | 35 | 48.4 |
| 11 | 40 | 5 | 3000 | 20 | 93.7 |
| 12 | 5 | 2 | 1900 | 20 | 58.7 |
| 13 | 21 | 8 | 1900 | 20 | 62.5 |
| 14 | 19 | 8 | 3000 | 35 | 63.3 |
| 15 | 17 | 8 | 800 | 35 | 49.3 |
| 16 | 31 | 2 | 800 | 35 | 36.1 |
| 17 | 8 | 8 | 1900 | 50 | 69.1 |
| 18 | 18 | 2 | 3000 | 35 | 92.0 |
| 19 | 26 | 5 | 800 | 50 | 35.8 |
| 20 | 36 | 8 | 1900 | 20 | 65.6 |
| 21 | 34 | 8 | 3000 | 35 | 46.1 |
| 22 | 12 | 5 | 3000 | 50 | 92.3 |
| 23 | 30 | 5 | 1900 | 35 | 93.4 |
| 24 | 22 | 2 | 1900 | 50 | 73.4 |
| 25 | 3 | 2 | 3000 | 35 | 91.7 |
| 26 | 42 | 5 | 3000 | 50 | 92.0 |
| 27 | 28 | 5 | 1900 | 35 | 96.0 |
| 28 | 10 | 5 | 3000 | 20 | 93.4 |
| 29 | 37 | 2 | 1900 | 50 | 77.1 |
| 30 | 13 | 5 | 1900 | 35 | 90.3 |
| 31 | 35 | 2 | 1900 | 20 | 66.8 |
| 32 | 20 | 2 | 1900 | 20 | 57.3 |
| 33 | 23 | 8 | 1900 | 50 | 80.8 |
| 34 | 38 | 8 | 1900 | 50 | 67.6 |
| 35 | 25 | 5 | 3000 | 20 | 93.1 |
| 36 | 43 | 5 | 1900 | 35 | 95.7 |
| 37 | 27 | 5 | 3000 | 50 | 93.7 |
| 38 | 24 | 5 | 800 | 20 | 69.3 |
| 39 | 29 | 5 | 1900 | 35 | 90.3 |
| 40 | 16 | 2 | 800 | 35 | 32.4 |
| 41 | 33 | 2 | 3000 | 35 | 91.7 |
| 42 | 32 | 8 | 800 | 35 | 60.2 |

| | | | | | |
|---|---|---|---|---|---|
| 43 | 41 | 5 | 800 | 50 | 48.4 |
| 44 | 14 | 5 | 1900 | 35 | 100.0 |
| 45 | 44 | 5 | 1900 | 35 | 90.3 |

b. Hempseed oil

| Sample number (Standard Order) | Randomized Order | Duration reaction (h) | Enzyme load (U) | Oil/aqueous ratio of the mixture (%) | Hydrolysis rate (%) |
|---|---|---|---|---|---|
| 1 | 38 | 8 | 1900 | 50 | 99.7 |
| 2 | 32 | 8 | 800 | 35 | 100.0 |
| 3 | 40 | 5 | 3000 | 20 | 98.6 |
| 4 | 36 | 8 | 1900 | 20 | 100.0 |
| 5 | 12 | 5 | 3000 | 50 | 75.1 |
| 6 | 15 | 5 | 1900 | 35 | 98.8 |
| 7 | 18 | 2 | 3000 | 35 | 69.9 |
| 8 | 31 | 2 | 800 | 35 | 39.1 |
| 9 | 6 | 8 | 1900 | 20 | 100.0 |
| 10 | 24 | 5 | 800 | 20 | 93.9 |
| 11 | 27 | 5 | 3000 | 50 | 100.0 |
| 12 | 45 | 5 | 1900 | 35 | 100.0 |
| 13 | 44 | 5 | 1900 | 35 | 100.0 |
| 14 | 5 | 2 | 1900 | 20 | 50.4 |
| 15 | 25 | 5 | 3000 | 20 | 100.0 |
| 16 | 7 | 2 | 1900 | 50 | 80.3 |
| 17 | 37 | 2 | 1900 | 50 | 73.3 |
| 18 | 42 | 5 | 3000 | 50 | 70.7 |
| 19 | 16 | 2 | 800 | 35 | 22.6 |
| 20 | 34 | 8 | 3000 | 35 | 77.1 |
| 21 | 14 | 5 | 1900 | 35 | 100.0 |
| 22 | 13 | 5 | 1900 | 35 | 93.9 |
| 23 | 4 | 8 | 3000 | 35 | 72.8 |
| 24 | 11 | 5 | 800 | 50 | 55.4 |
| 25 | 20 | 2 | 1900 | 20 | 50.4 |
| 26 | 9 | 5 | 800 | 20 | 83.5 |
| 27 | 26 | 5 | 800 | 50 | 60.9 |
| 28 | 28 | 5 | 1900 | 35 | 97.1 |
| 29 | 19 | 8 | 3000 | 35 | 71.6 |
| 30 | 2 | 8 | 800 | 35 | 95.9 |
| 31 | 23 | 8 | 1900 | 50 | 94.5 |
| 32 | 21 | 8 | 1900 | 20 | 100.0 |
| 33 | 10 | 5 | 3000 | 20 | 95.4 |
| 34 | 35 | 2 | 1900 | 20 | 48.7 |
| 35 | 30 | 5 | 1900 | 35 | 93.9 |
| 36 | 22 | 2 | 1900 | 50 | 69.9 |
| 37 | 3 | 2 | 3000 | 35 | 78.6 |
| 38 | 41 | 5 | 800 | 50 | 60.0 |
| 39 | 8 | 8 | 1900 | 50 | 95.1 |

| | | | | | |
|---|---|---|---|---|---|
| 40 | 1 | 2 | 800 | 35 | 32.8 |
| 41 | 33 | 2 | 3000 | 35 | 73.0 |
| 42 | 17 | 8 | 800 | 35 | 93.0 |
| 43 | 43 | 5 | 1900 | 35 | 100.0 |
| 44 | 39 | 5 | 800 | 20 | 91.0 |
| 45 | 29 | 5 | 1900 | 35 | 94.5 |

c. Linseed oil

| Sample number (Standard Order) | Randomized Order | Duration reaction (h) | Enzyme load (U) | Oil/aqueous ratio of the mixture (%) | Hydrolysis rate (%) |
|---|---|---|---|---|---|
| 1 | 35 | 2 | 1900 | 20 | 51.5 |
| 2 | 31 | 2 | 800 | 35 | 98.4 |
| 3 | 42 | 5 | 3000 | 50 | 74.0 |
| 4 | 40 | 5 | 3000 | 20 | 52.4 |
| 5 | 43 | 5 | 1900 | 35 | 87.8 |
| 6 | 33 | 2 | 3000 | 35 | 86.0 |
| 7 | 39 | 5 | 800 | 20 | 64.8 |
| 8 | 37 | 2 | 1900 | 50 | 66.2 |
| 9 | 44 | 5 | 1900 | 35 | 88.3 |
| 10 | 32 | 8 | 800 | 35 | 97.0 |
| 11 | 36 | 8 | 1900 | 20 | 80.0 |
| 12 | 38 | 8 | 1900 | 50 | 83.7 |
| 13 | 45 | 5 | 1900 | 35 | 91.0 |
| 14 | 41 | 5 | 800 | 50 | 83.7 |
| 15 | 34 | 8 | 3000 | 35 | 73.6 |
| 16 | 19 | 8 | 3000 | 35 | 75.0 |
| 17 | 25 | 5 | 3000 | 20 | 49.2 |
| 18 | 22 | 2 | 1900 | 50 | 65.3 |
| 19 | 20 | 2 | 1900 | 20 | 52.0 |
| 20 | 23 | 8 | 1900 | 50 | 92.6 |
| 21 | 26 | 5 | 800 | 50 | 84.6 |
| 22 | 29 | 5 | 1900 | 35 | 88.3 |
| 23 | 27 | 5 | 3000 | 50 | 72.7 |
| 24 | 28 | 5 | 1900 | 35 | 87.4 |
| 25 | 21 | 8 | 1900 | 20 | 77.7 |
| 26 | 17 | 8 | 800 | 35 | 99.3 |
| 27 | 18 | 2 | 3000 | 35 | 91.8 |
| 28 | 24 | 5 | 800 | 20 | 65.3 |
| 29 | 30 | 5 | 1900 | 35 | 85.1 |
| 30 | 16 | 2 | 800 | 35 | 100.0 |
| 31 | 13 | 5 | 1900 | 35 | 86.4 |
| 32 | 2 | 8 | 800 | 35 | 94.3 |
| 33 | 7 | 2 | 1900 | 50 | 65.3 |
| 34 | 11 | 5 | 800 | 50 | 84.1 |
| 35 | 5 | 2 | 1900 | 20 | 51.5 |
| 36 | 6 | 8 | 1900 | 20 | 70.1 |

| | | | | | |
|---|---|---|---|---|---|
| 37 | 14 | 5 | 1900 | 35 | 85.5 |
| 38 | 1 | 2 | 800 | 35 | 100.0 |
| 39 | 15 | 5 | 1900 | 35 | 86.9 |
| 40 | 10 | 5 | 3000 | 20 | 47.8 |
| 41 | 8 | 8 | 1900 | 50 | 85.5 |
| 42 | 4 | 8 | 3000 | 35 | 75.9 |
| 43 | 9 | 5 | 800 | 20 | 68.5 |
| 44 | 12 | 5 | 3000 | 50 | 73.6 |
| 45 | 3 | 2 | 3000 | 35 | 83.2 |

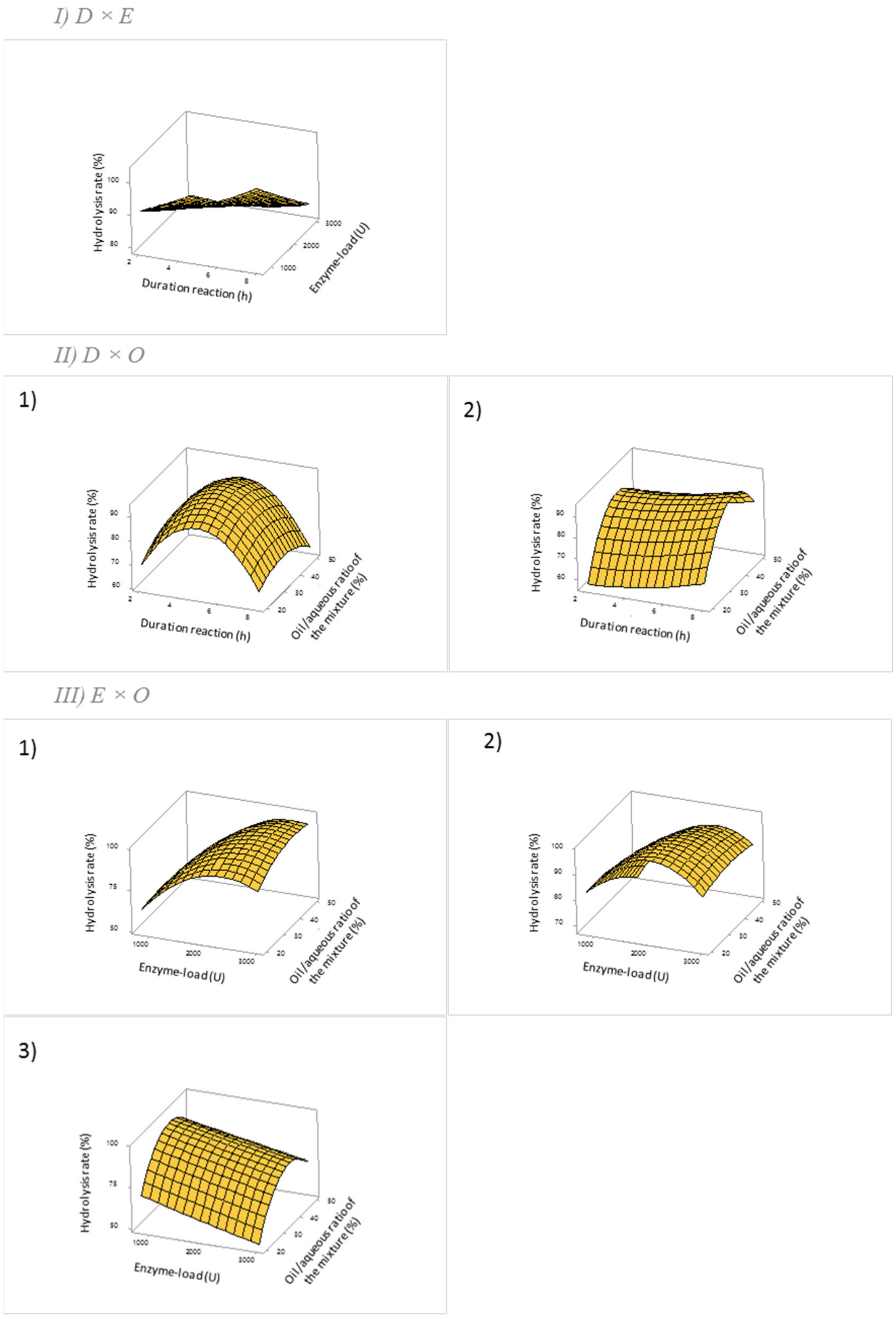


**Figure S3**. Three-dimensional (3D) response surface plots of the hydrolysis rate (%) with CRL, as function of : (I) the duration reaction (D) and enzyme-load (E) for linseed oil ; (II) the duration reaction (D) and oil/aqueous ratio of the mixture (O) for sunflower oil (1) and for linseed oil (2) ; (III) the enzyme-load (E) and oil/aqueous ratio of the mixture (O) for sunflower oil (1), hempseed oil (2) and linseed oil (3). The holding values are : 5 hours for the duration reaction, 1900U for the enzyme-load and 35% for the oil/aqueous ratio of the mixture.
